# A Variational Autoencoder Network for Heartbeat Signal Reconstruction and Breathing-State Classification Using 24 GHz Radar Sensors

**DOI:** 10.3390/s26217083

**Published:** 2026-11-09

**Authors:** Homa Arab, Nima Suzandeh, Serioja O. Tatu, Steven Dufour

**Affiliations:** 1 Department of Mathematics and Industrial Engineering, Polytechnique Montreal, Montreal, QC H3T 1J4, Canada; steven.dufour@polymtl.ca; 2 Institut National de la Recherche Scientifique (INRS), Energie Materiaux et Telecommunications, Montreal, QC H5A 1K6, Canada; nima.souzandeh@inrs.ca (N.S.); serioja.ovidiu.tatu@inrs.ca (S.O.T.)

**Keywords:** artificial intelligence, convolutional neural network (CNN), deep learning, denoising, Doppler frequency, machine learning, microwave, modulator, receiver, recurrent neural network (RNN), ResNet, signal classification, signal processing, variational autoencoder (VAE)

## Abstract

Wireless radar sensors can remotely detect vital signs. This study investigates the wireless monitoring of physiological signals induced by respiration and heartbeat to classify breathing states. Two 24 GHz radar sensors are used. Noise, harmonics, and intermodulation products affect the measured physiological signals and can make heartbeat identification and analysis challenging. Deep-learning techniques have recently been used to denoise and classify signals in wireless communications. Variational autoencoders (VAEs) have also been used for signal and image processing. Multilayer VAEs support sequential learning by optimizing generative-model parameters and reducing reconstruction error. This study investigates the separation of measured heartbeat and respiratory signals and the reduction in noise and the effects of limited random body movements using VAEs under controlled conditions. Two radars are positioned in front of and behind a human subject to record vital signals simultaneously. The separated and denoised heartbeat signals are input to a ResNet network to classify normal and fast breathing states. The proposed method achieves 99.7% accuracy on the reported experimental dataset.

## 1. Introduction

Radars can accurately detect a target’s Doppler frequency and wirelessly estimate its range [1,2]. Microwave radar sensors have proven to be accurate contactless sensors that can detect small movements and vibrations in the micrometer range [3]. They are used in applications such as non-contact vital-sign detection, sleep monitoring, human-fall detection, smart surveillance, and security [4,5]. They also offer greater flexibility than wearable sensors such as electrocardiogram (ECG) monitors, conventional photoplethysmography (PPG) sensors, and piezoresistive sensors.

Multiple-radar systems are attracting growing attention because they can simultaneously record different signals [6,7]. Recording several waveforms from a target enables the extraction of additional information for target detection and localization. In this work, we use two 24 GHz radar sensors to measure heartbeat and respiratory signals and classify normal and fast breathing states.

The proposed radar systems transmit orthogonal signals from two transmitter (TX) antennas, and the reflected signals are captured by the antennas of each receiver (RX). This setup makes it possible to collect long-term data from two receivers and detect small movements of one or more objects. Extracting the desired information from the received signal is an important and challenging task because of the various types of noise and interference found in RX signals, the nonstationary nature of the target, the existence of multiple targets, and the various RX antenna signals.

Various methods have been proposed to suppress noise and interference. Low-pass filters are commonly used to suppress noise in RF signals. Their implementation can be complex, and their parameters must be tuned to avoid attenuating low-frequency information. Machine-learning-based signal denoising has recently become popular in wireless communication systems, where these methods have shown strong performance in denoising and classification [8].

Recent studies applying deep-learning methods to heartbeat detection and classification are listed in Table 1. Most use ECG signals as input and convolutional neural networks (CNNs) or long short-term memory (LSTM) networks to classify raw time-domain signals or their scalograms. This study focuses on reconstructing heartbeat signals from physiological signals using VAEs. CNNs are also used for the final breathing-state classification. The VAE is a generative model used for signal and image processing, data augmentation, and generative modeling [9,10]. VAEs have the ability to provide meaningful latent representations of the input data produced by the encoder, and to generate new output signals from the compressed latent space via the decoder [11].

The contributions of this article can be summarized as follows:

- Two 24GHz radar sensors are used to measure the Doppler frequencies of a human subject’s physiological signals simultaneously. To the best of the authors’ knowledge, this is the first study to employ two radar sensors for this specific application. The sensors provide measurements from two directions at the same time. The radar measurement accuracy is assessed using metronome-based measurements. The mean frequency error is ±0.01Hz, and the mean range error is ±10micron [1].
- A VAE-based method is used to extract a heartbeat signal from a physiological signal and remove noise and interference from the heartbeat waveform. VAEs are powerful deep-learning techniques that have been used for signal and image processing. To the best of the authors’ knowledge, this is the first application of this method to heartbeat reconstruction and breathing-state classification.
- The reconstructed and denoised signals are input to a ResNet-50 neural network to classify normal and fast breathing states. The network achieves an accuracy of 99.7%.
- A shifting-based augmentation technique is used to double the dataset by shifting each sample by 1 ms along the time axis. The experimental results show that combining this augmentation technique with the ResNet-50 and VAE networks achieves 99.7% accuracy. The proposed technique may facilitate the extraction of essential physiological measures, such as breathing rate, pulse rate, body temperature, and blood pressure, from the denoised heartbeat signal.

## 2. Mathematical and Simulation Analyses

As illustrated in Figure 1, two identical commercial 24GHz CW Doppler radars are positioned in front of and behind a target. The block diagram and operating parameters of the radar are shown in Figure 1 and Table 2. The radar operates at a center frequency of 24GHz, has a bandwidth of 1GHz, and an equivalent isotropically radiated power (EIRP) of 30 dBm. It includes a low-noise microwave amplifier (LNA) to amplify the input signal. A balun at the receiver input converts the antenna’s unbalanced signal into the balanced differential signal required by the receiver or mixer. It also provides impedance matching and improves common-mode noise rejection. This compact, embedded radar can be integrated into various devices. A 24GHz microwave radar transmits a CW signal that can be written as

(1) $$T\left(t\right)=A_{\mathrm{TX}}\;\cos\;\left(2\pi ft+\theta (t)\right),$$

where θ(t) represents the phase shift created by the active components, $A_{\mathrm{TX}}$ is the amplitude level of the transmitted signal, and *f* is the center frequency. The target’s chest moves due to the heartbeat and the expansion and contraction of the lungs. Therefore, the signal received by the front radar contains information about both heartbeat and breathing rates. The radar behind the target, however, receives a reflected signal that contains only heartbeat information.

The reflected signal from the chest of the human target, denoted by R(t), can be written as

(2) $$R\left(t\right)=A_{\mathrm{RX}}\;\cos\;\left(2\pi f\left(t-t_{d}\right)+\theta \left(t-t_{d}\right)\right),$$

where $A_{\mathrm{RX}}$ is the received-signal amplitude, which depends on the transmitter and receiver antenna gains, the radar cross section, the target distance, and the carrier wavelength. The propagation delay, $t_{d}$, is given by

(3) $$t_{d}=\frac{2d(t)}{c}.$$

The time-varying distance between the radar and the target is

(4) $$d\left(t\right)=d_{0}+x\left(t\right),$$

where x(t) represents periodic chest movement, and $d_{0}$ is the distance between the radar and the target. Substituting Equations (3) and (4) into Equation (2), the received signal can be rewritten as

(5) $$R\left(t\right)=A_{\mathrm{RX}}\;\cos\;\left(2\pi ft-\left(\frac{4\pi d_{0}\;+\;4\pi x\left(t\right)}{\lambda }\right)+\theta \left(t-\frac{2d(t)}{c}\right)\right).$$

The baseband in-phase (*I*) and quadrature-phase (*Q*) signals are given by:

(6) $$\begin{array}{c} I\left(t\right)=A_{I}\cos\;\left(\left(\frac{4\pi d_{0}\;+\;4\pi x\left(t\right)}{\lambda }\right)+\theta \left(t-\frac{2d(t)}{c}\right)\right);\\ Q\left(t\right)=A_{Q}\sin\;\left(\left(\frac{4\pi d_{0}\;+\;4\pi x\left(t\right)}{\lambda }\right)+\theta \left(t-\frac{2d(t)}{c}\right)\right), \end{array}$$

where $A_{I}$ and $A_{Q}$ are the amplitudes of the *I* and *Q* signals, respectively. The residual phase noise $\theta \left(t\right)-\theta \left(t-2d(t)/c\right)$ is negligible because the sensor is coherent. The TX and local oscillator (LO) signals share a source and phase noise, which can therefore cancel. The chest displacement x(t) can be modeled as a function of the breathing and heartbeat rates:

(7) $$\begin{array}{c} x\left(t\right)=x_{r}\left(t\right)+x_{h}\left(t\right);\\ x\left(t\right)=m_{r}\cos\left(2\pi f_{r}t\right)+m_{h}\cos\left(2\pi f_{h}t\right), \end{array}$$

where $x_{r}\left(t\right)$ and $x_{h}\left(t\right)$ are the chest displacements due to respiration and heartbeat, respectively. The quantities $m_{r}$ and $m_{h}$ are their displacement magnitudes, while $f_{r}$ and $f_{h}$ are their frequencies. Substituting Equation (7) into Equation (6), the baseband in-phase (*I*) and quadrature-phase (*Q*) signals can be written as

(8) $$\begin{array}{c} I\left(t\right)=A_{I}\cos\left[\frac{4\pi x_{r}\left(t\right)}{\lambda }+\frac{4\pi x_{h}\left(t\right)}{\lambda }+\frac{4\pi d_{0}}{\lambda }\right];\\ Q\left(t\right)=A_{Q}\sin\left[\frac{4\pi x_{r}\left(t\right)}{\lambda }+\frac{4\pi x_{h}\left(t\right)}{\lambda }+\frac{4\pi d_{0}}{\lambda }\right]. \end{array}$$

To analyze the harmonics and frequency components of the received radar signal, we use a Fourier-series expansion involving Bessel functions $J_{i}\left(x\right)$ of order *i*. Equation (9) shows that respiratory harmonics and intermodulation products between the respiratory and cardiac signals can affect detection accuracy. The heartbeat peak amplitude depends on both $m_{r}$ and $m_{h}$; it corresponds to the terms (k=0,l=±1).

(9) $$\begin{array}{c} I\left(t\right)=\sum_{k=-\infty }^{+\infty }\sum_{l=-\infty }^{+\infty }J_{k}\left(\frac{4\pi m_{r}}{\lambda }\right)J_{l}\left(\frac{4\pi m_{h}}{\lambda }\right)\\ \;\;\;\;\;\;\;\;\;\;\;\;\;\;\cos\left(2\pi (kf_{r}+lf_{h})t\right)\cos\left(\frac{4\pi d_{0}}{\lambda }\right);\\ Q\left(t\right)=\sum_{k=-\infty }^{+\infty }\sum_{l=-\infty }^{+\infty }J_{k}\left(\frac{4\pi m_{r}}{\lambda }\right)J_{l}\left(\frac{4\pi m_{h}}{\lambda }\right)\\ \;\;\;\;\;\;\;\;\;\;\;\;\;\;\sin\left(2\pi (kf_{r}+lf_{h})t\right)\sin\left(\frac{4\pi d_{0}}{\lambda }\right). \end{array}$$

As mentioned earlier, the reflected signal from behind the target contains only heartbeat information. Therefore, the baseband in-phase I and quadrature-phase Q can be written as:

(10) $$\begin{array}{c} I\left(t\right)=\sum_{l=-\infty }^{+\infty }J_{l}\left(\frac{4\pi m_{h}}{\lambda }\right)\cos\left(2\pi lf_{h}t\right)\cos\left(\frac{4\pi d_{0}}{\lambda }\right);\\ Q\left(t\right)=\sum_{l=-\infty }^{+\infty }J_{l}\left(\frac{4\pi m_{h}}{\lambda }\right)\sin\left(2\pi lf_{h}t\right)\sin\left(\frac{4\pi d_{0}}{\lambda }\right). \end{array}$$

In the experimental setup shown in Figure 1, a Tektronix DPO7054 oscilloscope displays the amplitude, phase, and period of the received baseband *I* and *Q* signals. An Agilent DSO-X 2024A oscilloscope provides a second display for the Lissajous curve in the *I*/*Q* plane. The direction of motion along a circle or spiral in this plane (clockwise or counterclockwise) depends on the direction of the target’s chest movement. A DC source provides a tuning voltage between 0 and 5 V to adjust the frequency across the 1 GHz bandwidth, from 23.5 GHz to 24.5 GHz. A TFC-3000 digital universal frequency counter measures and displays the transmitted-signal frequency; the displayed value is multiplied by 8192. The setup uses simple, low-cost components.

Figure 2 shows the captured in-phase baseband signals in the time and frequency domains for several conditions: normal breathing, fast breathing, and breath-holding for 30 s. The waveforms were recorded by radar sensors positioned in front of and behind a human subject. Their spectra show the respiration frequency and its first three harmonics, intermodulation components, and the heartbeat frequency. In the next section, we describe a deep generative network for extracting relevant features from the recorded signals. A ResNet-50 CNN is used to classify normal and fast breathing states.

The present evaluation was conducted under controlled laboratory conditions and included only limited random body shifts. Minor changes in posture or target position can alter the radar-target distance, angle, phase, and signal amplitude, which may affect VAE reconstruction and heartbeat–respiration separation. The VAE can therefore reduce the effects of the limited movements observed in this setup, but the current study does not provide quantitative results for fully unconstrained movement. A systematic evaluation using predefined positional shifts and reconstruction, signal-to-noise, and classification metrics is left for future work.

## 3. Variational Autoencoders (VAEs)

A variational autoencoder (VAE) is a deep generative model introduced by Diederik P. Kingma and Max Welling in 2013 [22]. It has an architecture similar to that of a standard autoencoder, but differs in its mathematical formulation and the distributions used in its latent space. These properties reduce discontinuities in the latent representation and improve the model’s ability to generate outputs. VAEs have been used in image generation [23], text generation [24], learning basic visual concepts [25], and speech-spectrogram modeling [26].

A VAE has three components: a probabilistic encoder, a sampling unit, and a decoder. The basic architecture of a VAE is shown in Figure 3.

The encoder, or recognition model, comprises convolutional networks that take the input (**x**) and map it to a lower-dimensional latent representation (*z*). The marginal likelihood is

$$\begin{array}{c} p_{\theta }\left(x\right)=\int p_{\theta }\left(z\right)p_{\theta }\left(x|z\right)\;dz. \end{array}$$

The encoder approximates the true posterior distribution, $p_{\theta }\left(z|x\right)$, using $q_{\phi }\left(z|x\right)$. This approximate posterior is assumed to be Gaussian, and ϕ denotes the encoder’s variational parameters. The encoder outputs the mean and standard deviation of $q_{\phi }\left(z|x\right)$. The decoder, denoted by $p_{\theta }\left(x|z\right)$, attempts to reconstruct the original input from the latent representation *z*. A sampling unit draws the latent vector *z* from the approximate posterior as follows:

(11) $$\begin{array}{c} z∼q_{\phi }\left(z|x\right)=N\left(\mu ,\sigma \right) \end{array}$$

where μ is the mean vector and σ is the standard-deviation vector.

The variational parameters ϕ and generative-distribution parameters θ are learned by training the VAE. The loss function to be minimized has two components: a reconstruction loss and a regularization loss, given by

The VAE architecture used in this work is shown in Figure 4. The VAE receives two time-domain input signals. Before augmentation, the raw VAE dataset contained 15,000 samples. It was divided into 10,000 training samples (66.7%), 2500 validation samples (16.7%), and 2500 testing samples (16.7%). All samples came from a single participant (n=1), and no k-fold cross-validation was performed. Thus, these results demonstrate VAE feasibility under the considered experimental conditions, but do not establish subject-independent generalizability. The first input signal contains heartbeat and respiratory components recorded by the front radar. The second contains the heartbeat waveform recorded by the radar behind the target. Each input column contains 100,000 points, which are reduced to 10,000 by averaging each consecutive set of 10 points. This factor-of-10 reduction was selected to balance signal preservation and computational cost. Averaging consecutive samples attenuates rapid sample-to-sample fluctuations before decimation, thereby reducing high-frequency noise and the memory and computational requirements of the VAE. Because the heartbeat and respiratory components are lower-frequency physiological signals relative to the original acquisition rate, the procedure is intended to retain their temporal waveforms, including the small amplitude variations associated with heartbeat micro-movements, while suppressing unwanted high-frequency fluctuations. The 10,000 points are divided into windows of 256 points with a stride of 8. The batch size is 60. As shown in Figure 4, the encoder uses four convolutional layers with 32, 64, 128, and 256 channels, each with a kernel size of 3. After encoding, the signal is flattened into a one-dimensional feature vector used to compute the mean and variance of the latent distribution. These parameters are used to sample the latent space; the resulting samples are passed to the decoder to reconstruct the heartbeat and respiratory signals.

(12) $$\begin{array}{c} L\left(\theta ,\phi \right)=-E_{z∼q_{\phi }\left(z|x\right)}\left(\log p\left(\tilde{x}|z\right)\right)+D_{KL}\left(q_{\phi }\left(z|x\right)∥p_{\theta }\left(z\right)\right) \end{array}$$

where L denotes the VAE loss associated with the encoder and decoder parameters. The first term is the reconstruction term, which measures the discrepancy between the input and its reconstruction. The second term, $D_{KL}\left(\cdot \right)$, is the Kullback–Leibler (KL) divergence between the approximate posterior and the latent-variable prior. Minimizing this term regularizes the approximate posterior toward the prior distribution.

(13) $$\begin{array}{c} D_{KL}\left(q_{\phi }\left(z|x\right)∥p_{\theta }\left(z\right)\right)\\ =\frac{1}{2}\sum_{j=1}^{J}\left(\mu_{j}^{2}+\sigma_{j}^{2}-1-\log\sigma_{j}^{2}\right). \end{array}$$

Further details on the derivation of the VAE loss are provided in [9,10]. All convolutional layers use the LeakyReLU activation function, and 30% dropout is used to help prevent overfitting. Each layer also includes a batch-normalization layer to reduce training time and improve stability. The encoder output is flattened into a feature vector used to compute the mean and variance of the latent space. The decoder comprises four transposed-convolution layers and one convolutional layer. The Adam optimizer, with $\beta_{1}=0.9$ and $\beta_{2}=0.999$, is used for training.

A learning-rate scheduler with a step size of 40 epochs and a gamma of 0.9 is used during training. Figure 5a shows the signal recorded by the front radar. Figure 5b,c show the VAE output signals corresponding to respiration and heartbeat, respectively. The denoised and separated heartbeat signal is then input to a ResNet network for classification of normal and fast breathing states.

### ResNet Convolutional Networks

Convolutional neural networks (CNNs) are deep-learning methods for classification and feature extraction from many types of data. Their use in image and signal processing has grown substantially in recent decades. To improve classification and estimation accuracy, some researchers use CNNs with more layers, known as deep convolutional neural networks (DCNNs). Adding convolutional layers can improve classification accuracy until the network saturates; beyond that point, accuracy may decrease, and overfitting and training error may increase [27]. Vanishing gradients can also arise in DCNNs and may prevent the weights from converging during backpropagation. He et al. introduced residual neural networks (ResNets) to address vanishing-gradient and saturation problems [28]. The ResNet-50 architecture used in this work is shown in Figure 6, and its bottleneck block is shown in Figure 7.

ResNet uses identity connections between layers to pass input data directly to deeper layers.

Skip connections allow information to pass through deeper layers and help the network learn identity mappings. The residual mapping, F(x), represents the difference between the input and output of a residual block. The residual mapping is added to the block input to produce the block output, H(x). In training the residual network, the main focus is finding and minimizing F(x). After finding F(x), the deeper layers can also produce extra feature maps.

Due to laboratory-related limitations, measurements were conducted under two breathing conditions: normal and fast breathing. The input dataset comprises 250 baseband I/Q signals across these two classes. All recorded signals are from a single participant (n=1). Age, gender, health status, positioning variability, and subject-to-subject variability were not assessed; therefore, the results are not generalizable to other individuals or populations. The ResNet-50 is applied to the I/Q signals collected by the 24 GHz radar sensors positioned in front of and behind the subject, after VAE-based separation of the respiratory components. Data augmentation is used to expand the training dataset and help reduce overfitting. In this work, a shifting-based augmentation technique doubles the dataset by shifting each sample by 1 ms along the time axis.

The Adam optimizer with a learning rate of 0.001 and a cross-entropy loss function was used for classification. The ResNet-50 achieved a classification accuracy of 99.7% after 100 training epochs. A conventional CNN achieved an accuracy of 96.5% under the reported experimental conditions.

The proposed VAE ResNet-50 pipeline achieved a 3.2 percentage-point improvement in breathing-state classification accuracy over the conventional CNN under the same reported experimental conditions. The VAE is designed to learn an adaptive nonlinear mapping from the paired front- and rear-radar signals, enabling noise and motion-artifact suppression while reconstructing the heartbeat and respiratory components. In contrast, conventional low-pass, band-pass, or wavelet filtering is primarily frequency-based and can attenuate desired vital-sign components when they overlap spectrally with respiratory harmonics, motion artifacts, or intermodulation products. Learning-based denoisers can also provide adaptive suppression, but their performance depends on the training objective, architecture, and availability of representative paired noisy and clean signals. The current accuracy comparison evaluates the end-to-end classification pipeline and does not isolate the VAE contribution.

Figure 8 presents the confusion matrices for the two networks. They show the numbers of false positives, false negatives, true positives, and true negatives for normal and fast breathing. In the displayed results, the ResNet-50 correctly classifies both classes with no errors. Future work will use the extracted heartbeat signal to estimate additional physiological measures.

## 4. Conclusions

This study investigated the wireless monitoring of physiological signals induced by respiration and heartbeat using two microwave radar sensors. A variational autoencoder (VAE) network was proposed to separate the heartbeat signal from the respiratory signal and denoise the heartbeat component. Under controlled conditions, the method reduced the effects of noise and limited random body movement in the measured heartbeat signal. A ResNet-50 network was then used to classify the denoised heartbeat signal. The proposed method achieved an overall accuracy of 99.7% in classifying normal versus fast breathing. Further work may investigate estimating physiological measures, including heart rate, breathing rate, temperature, and blood pressure, from the denoised heartbeat signal.

## Figures and Tables

**Figure 1:**
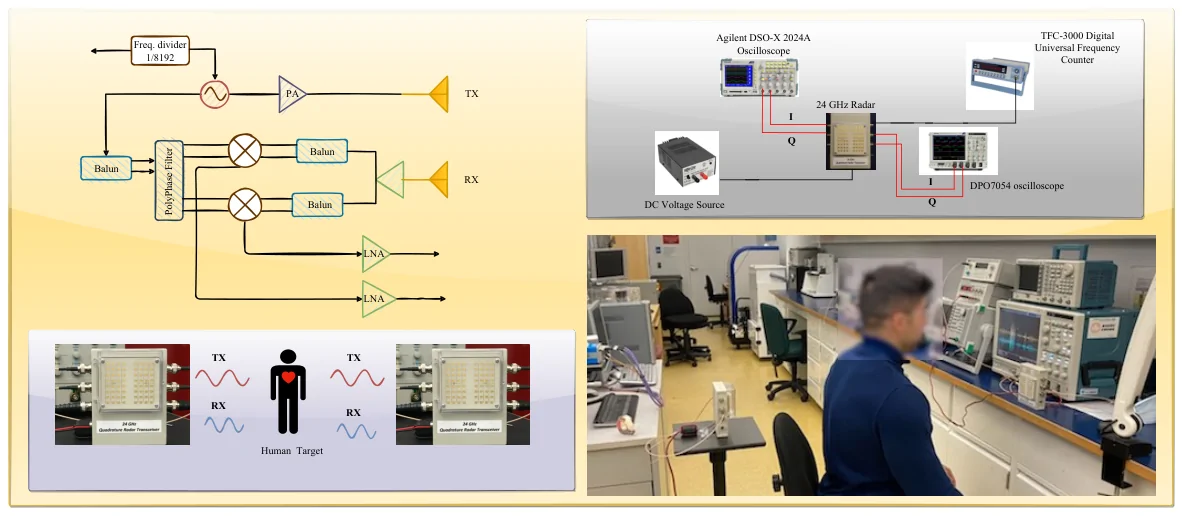
Block diagram and experimental setup for measuring heartbeat and respiration using two 24 GHz CW radars positioned in front of and behind a human subject.

**Figure 2:**
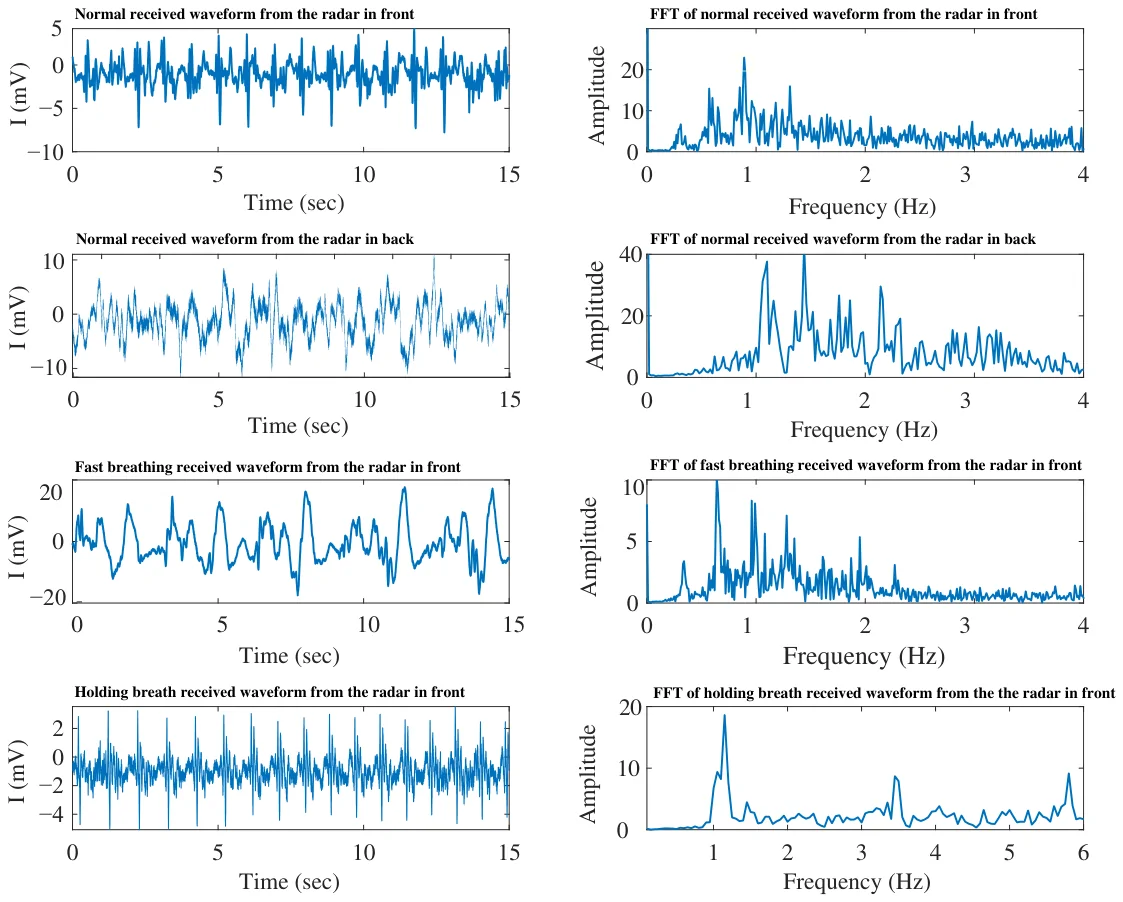
Waveform and spectrum of received signal in various states.

**Figure 3:**
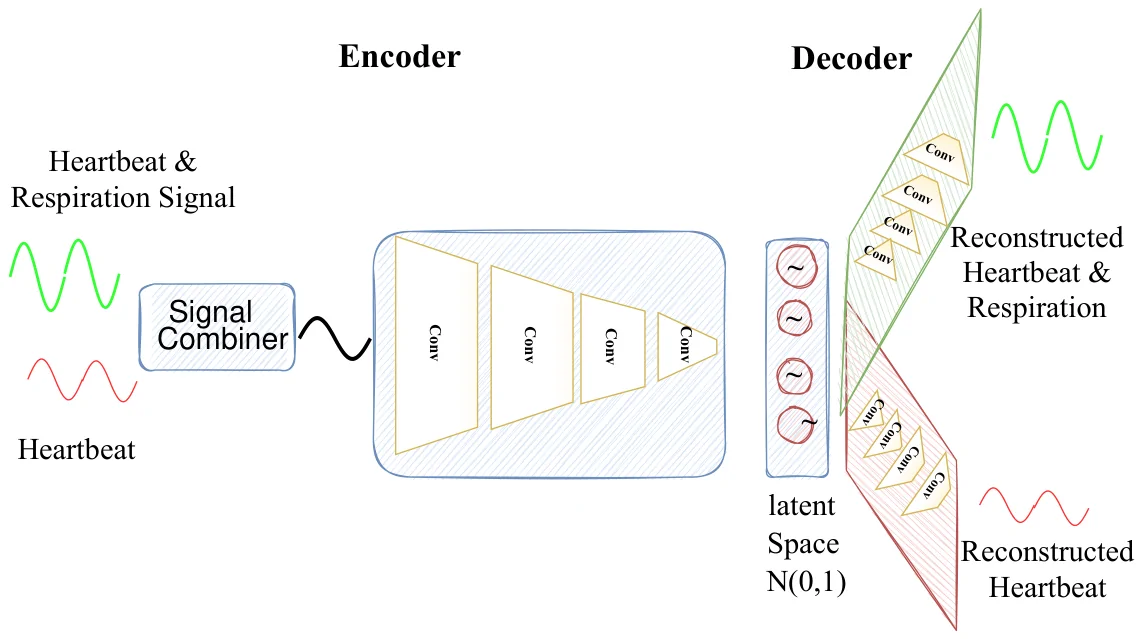
Architecture of the proposed VAE network.

**Figure 4:**
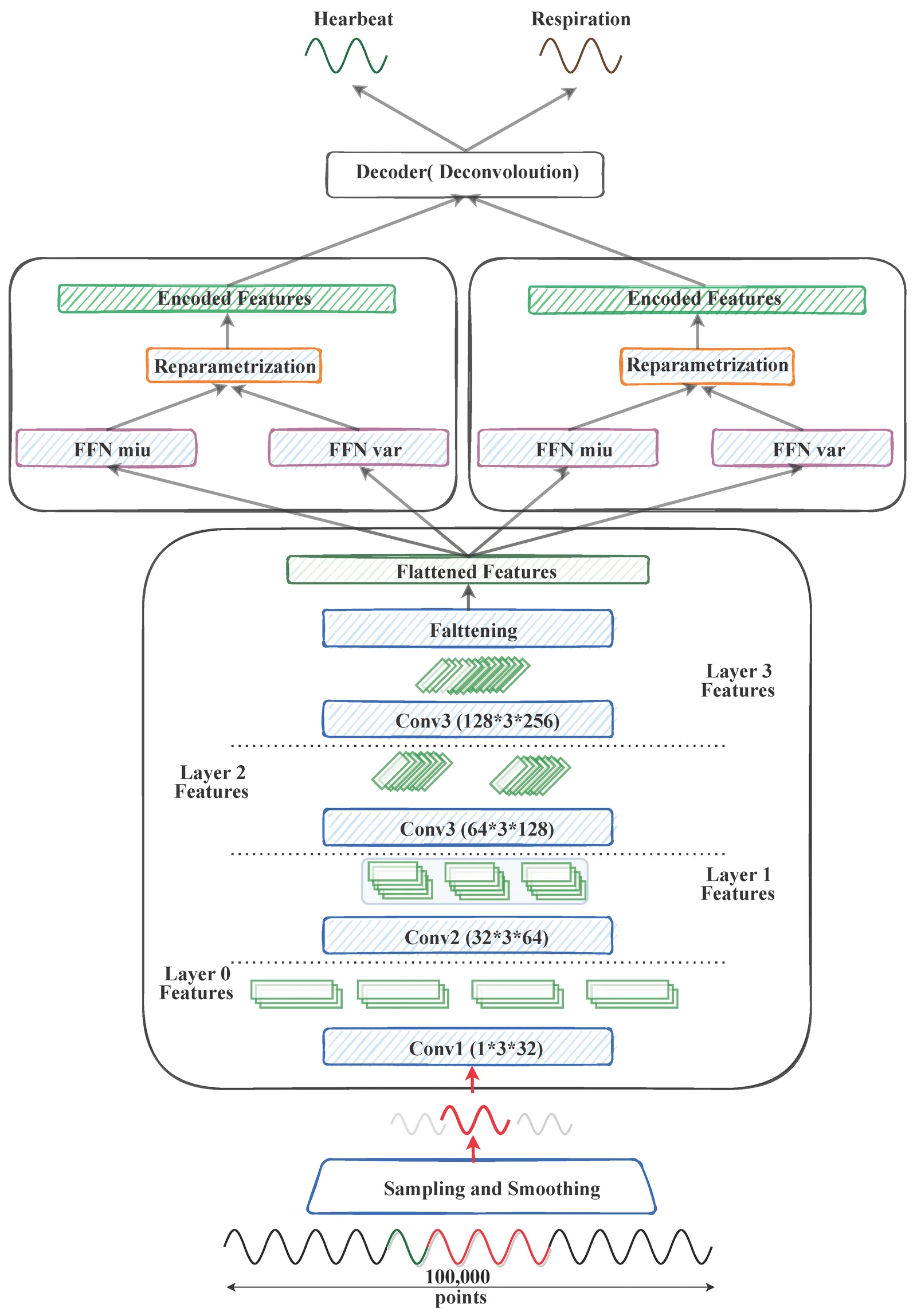
Block diagram Block diagram and key details of the VAE used in this study.

**Figure 5:**
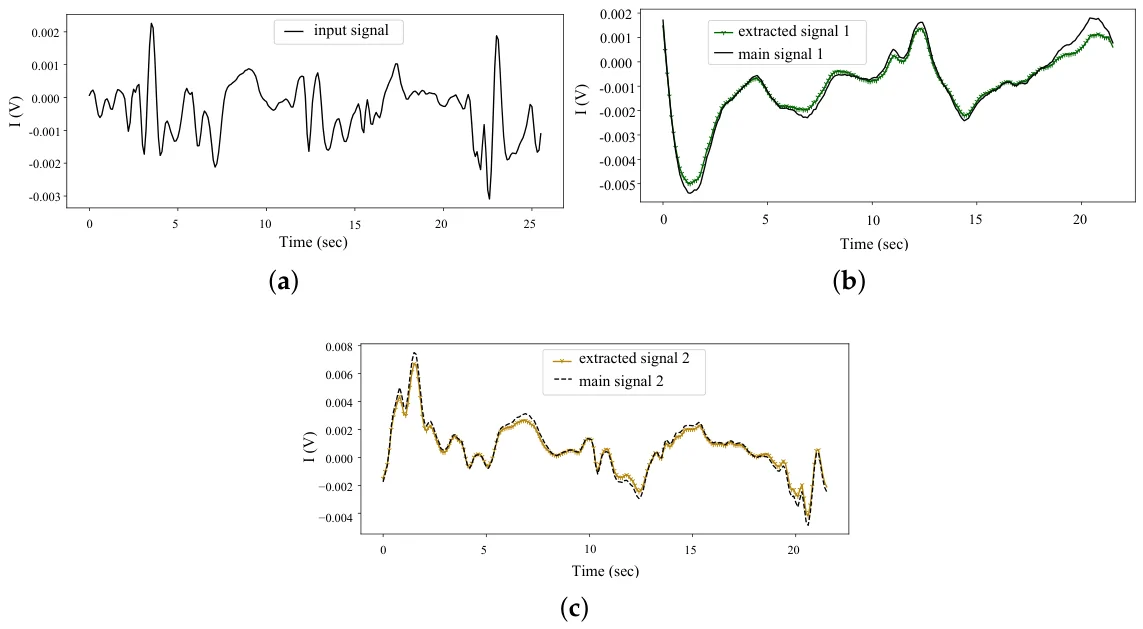
Input and output signals of the VAE network. The input contains heartbeat and respiratory components; the outputs represent respiration (signal 1) and heartbeat (signal 2). (**a**) The VAE input signal includes heartbeat and respiratory signals; (**b**) The VAE output signal represents the respiratory signal; (**c**) The VAE output signal represents the heartbeat signal.

**Figure 6:**
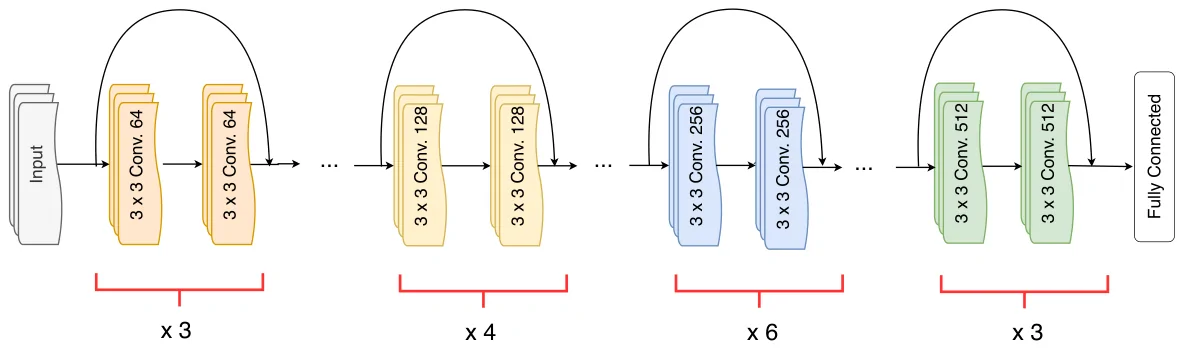
Architecture diagram of ResNet-50.

**Figure 7:**
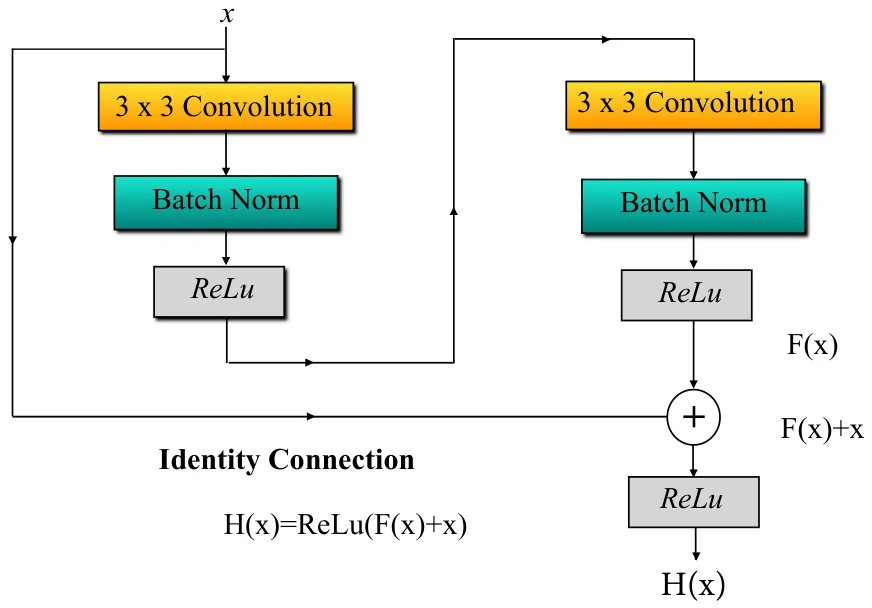
A bottleneck block of a residual network with two layers.

**Figure 8:**
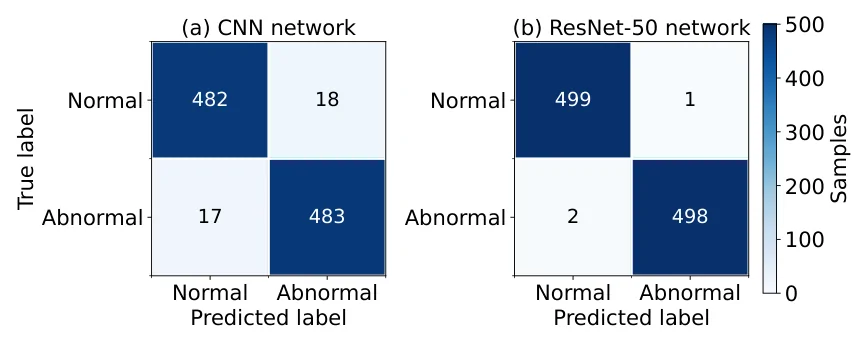
Confusion matrices for the system using (**a**) a CNN and (**b**) ResNet-50.

**Table 1:** A survey of machine learning neural network applications for heartbeat signal reconstruction and classification.

Ref.	Instrument	Machine Learning Technique	Accuracy	Application
[12]	Electrocardiogram (ECG)	MPCNN	96.4%	Inter-Patient ECG Classification
[13]	Electrocardiogram (ECG)	CNN	98.45%	Heartbeat ECG Classification
[14]	Electrocardiogram (ECG)	ELM-LRF and BLSTM	99.32% and 97.15%	Arrhythmia Detection
[15]	Electrocardiogram (ECG)	ResNet-101	99.75%	Heartbeat ECG Classification
[16]	MIT-BIH Database	CNN/LSTM	98%	Heartbeat Detection
[17]	Doppler radar 24 GHz	LSTM	NA	Heartbeat Signal Reconstruction
[18]	CW Doppler Radar 2.4 GHz	CNN/LSTM	86.6%	Sleep Stage Estimation
[19]	Doppler radar 5.8 GHz	ConceFT algorithm	NA	Heart-Rate Detection
[20]	CW Doppler Radar 2.4 GHz	SVM, LDA, and NB	88%, 86%, and 80%	Vital Signs Monitoring
Proposed	Two CW Doppler Radars, 24 GHz	Variational Autoencoders (VAEs); ResNet Convolutional Networks	99.7%	Heartbeat Signal Reconstruction and Breathing-State Classification

**Table 2:** Characteristics of the proposed radar [21].

Characteristic	Value
Center frequency	24 GHz
Frequency bandwidth deviation	1 GHz (23.5 to 24.5 GHz)
Horizontal & vertical −3 dB beamwidth	24° & 16°
Side lobe level (*H*-plane)	−29 dB
Side lobe level (*E*-plane)	−11 dB
TX & RX antenna gain	16 dBi
First IF LNA	20 dB
Second IF LNA	14 dB
Ambient temperature range	[−25 °C, 85 °C]
Storage temperature range	[−40 °C, 105 °C]

## Data Availability

Data is available on request from the corresponding author.
